# Using theory and formative research to design interventions to improve community health worker motivation, retention and performance in Mozambique and Uganda

**DOI:** 10.1186/s12960-015-0020-8

**Published:** 2015-04-30

**Authors:** Daniel Llywelyn Strachan, Karin Källander, Maureen Nakirunda, Sozinho Ndima, Abel Muiambo, Zelee Hill, the inSCALE study group

**Affiliations:** UCL Institute for Global Health, 30 Guilford St., London, WC1N 1EH UK; Malaria Consortium, Development House, 56-64 Leonard Street, London, EC2A 4LT UK; Karolinska Institutet, Stockholm, Sweden; Malaria Consortium Uganda, Plot 25 Upper Naguru East Road, P.O.Box 8045, Kampala, Uganda; Malaria Consortium Mozambique, Rua Joseph Ki-Zerbo 191, PO Box 3655, Coop, Maputo Mozambique

**Keywords:** Community health workers, Motivation, Retention, Performance, Social Identity Approach, Human resources for health, Uganda, Mozambique

## Abstract

**Background:**

Community health workers (CHWs) are increasingly being used in low-income countries to address human resources shortages, yet there remain few effective, evidence-based strategies for addressing the enduring programmatic constraints of worker motivation, retention and performance. This paper describes how two interventions were designed by the Innovations at Scale for Community Access and Lasting Effects (inSCALE) project to address these constraints in Uganda and Mozambique drawing on behavioural theory and formative research results.

**Methods:**

A review of the work motivation and CHW motivation literature—incorporating influences on retention and performance—was conducted on articles sourced through electronic web searches. Formative research with a focus on the barriers and facilitators to CHW motivation, retention and performance was conducted with community health workers and key stakeholders in Uganda and Mozambique. An analytical induction approach to the thematic analysis of transcripts from 98 in-depth interviews and 26 focus group discussions was adopted across the country settings.

**Results:**

From the theoretical review, it was determined that the interventions should promote CHWs as members of a collective by highlighting a sense of shared experience, focus on alignment between worker and programme goals, and emphasise the actions that lead to good performance. The Social Identity Approach was selected as the theory most likely to lead to the development of effective, scalable and sustainable interventions by addressing the identified gap in the literature of the influence of CHW working context. The formative research indicated that CHWs value feedback and feeling connected to the health system and their community, are motivated by status and community standing, and want to be provided with the necessary tools to perform. Two interventions based on these results were developed: a participatory, local community approach and an information communication technology (ICT) approach.

**Conclusions:**

Drawing on contextual data and theory that is sensitive to context can potentially lead to the development of appropriate and effective interventions when aiming to improve the motivation, retention and performance of CHWs in Uganda and Mozambique and other comparable settings. Evaluation of the developed interventions is crucial to assess this potential.

## Background

Increasingly, community health workers (CHWs) are being utilised in low-income countries to perform key health tasks and address human resources shortages in line with the WHO’s ‘task shifting’ agenda [1-8]. Despite this trend, the motivation, retention and performance of CHWs remain key reported constraints to the success of programmes in these settings [5,9]. While there is little available evidence relating to effective interventions to address these constraints [9-12], basing behavioural interventions on a theoretical understanding of behaviour, and empirical data from the behavioural context, has recently been emphasised when seeking to design successful public health programmes [4,9,13-15].

Previous enquiry focusing on the motivation, retention and performance of cadres of CHWs has typically sought to determine the right incentives to elicit tangible improvements in these outcomes [10,11,16-19]. In the few examples where theory has been drawn on to understand these outcomes, it has tended to focus on processes within the individual, with social and community factors largely providing contextual description [12,16,20]. Guidelines for managers of CHW programmes often recommend an approach that incorporates health system and community level factors [9,10,19]; however, psychological explanation of how such approaches may or may not lead to improved motivation, retention and performance remains dominated by behavioural theories that focus on the individual [13,21]. From a social psychological perspective, the importance of understanding the role of community context and culture, as well as individual cognitions, has been emphasised when seeking insight into the factors influencing CHW motivation, retention and performance [4,9].

The aim of this paper is to demonstrate how a behavioural theory, which accounts for the influence of group identification, in combination with data generated from qualitative interviews with CHWs and stakeholders, can be used to inform the design of interventions to improve CHW motivation, retention and performance in two settings—Uganda and Mozambique—with diverse, government-led CHW programmes. This work was conducted as part of the Innovations at Scale for Community Access and Lasting Effects (inSCALE) project [22,23], which is testing the impact of the interventions on the motivation, retention and performance of CHWs delivering Integrated Community Case Management (ICCM) of childhood diseases in Uganda and Mozambique. The inSCALE project will advocate for the incorporation of interventions found to be cost effective into the national CHW strategies of the respective countries.

## Methods

Several methods were used to design the interventions including drawing on reviews of the implementation context, stakeholder consultations, theoretical reviews and formative research. A description of this process appears in Table 1, with more detailed accounts reported elsewhere [23,24]. This paper reports on two critical components of the process: the review of theory and the formative research.

**Table 1:** **Process of identification and development of interventions**

**Step**	**Process**
1	Identify interventions with the potential to improve the motivation, retention and performance of CHWs in Uganda and Mozambique by reviewing theoretical and empirical evidence, consulting with key stakeholders in the field and exploring the political and programmatic operating context
2	Conduct formative research with the key personnel targeted by and tasked with implementation of the proposed interventions to explore their feasibility and acceptability. Use the data generated to reduce the number of possible interventions
3	With the same personnel, explore the barriers and facilitators to CHW motivation, retention and performance and incorporate these lessons into intervention design
4	Design interventions to be implemented in Uganda and Mozambique and their implementation strategies drawing on theoretical and empirical evidence and the formative research data

### Review of theory

We conducted reviews of theoretical literature relating to work motivation, retention and performance in general and to CHW motivation, retention and performance. Electronic internet searches on PubMed, Google and Google Scholar were performed using combinations of the following search terms: ‘motivation’, ‘retention’, ‘performance’, ‘work’, ‘health worker’, ‘community health worker, ‘CHW’, ‘community based agent’ and ‘CBA’. Relevant websites, such as www.chwcentral.org, were hand searched, as were the reference lists of included resources. Inclusion criteria were that studies be reported in the English language and, in the case of CHW motivation, published since 1990. Key CHW motivation resources were also identified through correspondence with authors who have published in the area.

We used this review to identify key theoretical dimensions to consider during intervention design and to select a focus theory. The selection of the focus theory to draw on was based on an assessment by the inSCALE study group of which was most likely to assist the development of effective interventions that would increase CHW motivation, retention and performance in the study context. Once the most suitable theory was selected, a second search was conducted using the terms ‘social identity’ and ‘social identification’.

### Formative research

#### Study setting

The study was conducted in Uganda’s capital, Kampala, as well as in two districts (Kiboga and Hoima) in the country’s mid-western region in January, February and May 2011 and in Massinga District and Inhambane Town in the central province of Inhambane, Mozambique, as well as in the capital city of Maputo in March 2012. The areas of operation were selected by the inSCALE project based on the ability to control for drug supply—a key additional programmatic constraint to health-focused CHW programmes—as these were provided by other projects. In both countries, the Ministries of Health utilise CHWs to deliver health promotion and ICCM of childhood diseases (namely malaria, pneumonia and diarrhoea). In keeping with the diverse nature of CHW programmes across country settings [25], there are however key programmatic differences between Uganda and Mozambique relating to CHW training, remuneration and support. In Uganda, CHWs are known as Village Health Team members (VHTs), operate as part of a five-member team and receive between 6 and 11 days training with between 25 and 90 CHWs per supervisor [26]. In Mozambique, CHWs are known as Agente Polivalente Elementares (APEs), work alone in their community and receive 4 months training with 2–3 CHWs per supervisor [27]. In Uganda, CHWs receive a travel allowance of US$ 4 per month and in Mozambique a subsidy of US$ 40 that represents approximately 60% of a minimum salary. While funding for the CHW programmes comes from external donors, in both countries, the role of NGOs has increasingly moved from implementation to technical support and quality assurance. The management and supply for all commodities and coordination of implementation is run by the respective national governments and district (Uganda) and provincial (Mozambique) authorities. The consistency of commodity supply is often however, as noted above, subject to donor assistance.

#### Sampling

In-depth interviews (IDIs) and focus group discussions (FGDs) were conducted with a range of target groups in Uganda and Mozambique. The respondent groups, methods and number and content of these research encounters are presented in Table 2. Sampling was purposive and for CHWs was based on stratification by relative proximity to supervising facility, use of mobile phones and network coverage as well as size of their community. Caregivers, mothers of children below 5 years and male heads of household were also stratified by size of community and proximity to a health facility. Supervisors were stratified by size of community and network coverage. Interview numbers were set in order to recruit multiple respondents in each stratum and maximise the likelihood of data saturation. Provision was made to recruit additional respondents in the event that data saturation was not reached, but this was not required.

**Table 2:** **Respondent groups, methods and number and content of research encounters**

**Country**	**Method**	**Respondents**	**Content**
	**(number of encounters)**		
Uganda	IDIs (5), FGDs (3)	Ministry of Health personnel at national and district levels	Ranking of a long list of possible interventions and discussion of their acceptability and feasibility in context
Mozambique	IDIs (6), FGDs (4)	Ministry of Health personnel at national, district and provincial levels	
Uganda	IDIs (61)	CHWs (31), CHW supervisors (6), NGO and district personnel with experience in CHW programme implementation (6), local community leaders (6), caregivers of children below 5 years (6) and male heads of household (6)	CHW motivation and issues related to their retention, performance and interaction with their community Acceptability and feasibility of possible interventions
Mozambique	IDIs (26)	CHWs (12), supervisors (6), community leaders (4), district- and province-level personnel with experience in CHW programme implementation (4)	
Uganda	FGDs (15)	CHWs (7), supervisors (3), district personnel with experience in CHW programme implementation (2), local community leaders (1), caregivers of children below 5 years (1) and male heads of household (1)	Acceptability and feasibility of possible interventions
Mozambique	FGDs (4)	Mothers of children below 5 years (4)	

#### Data collection and analysis

IDIs and FGDs ranging in duration from 45 to 120 min were conducted and digitally recorded in local languages and English by trained, multi-lingual fieldworkers using pre-tested discussion guides. Informed consent of participants was taken in compliance with the ethical approval conditions of the project^a^ [28]. Audio recordings and fieldworker notes were used to produce *expanded notes* from what the respondent or respondents said with direct quotations used to illustrate the main points [29,30]. These were produced immediately following the research encounter and before the next interview or FGD to allow for more accurate capture of the content while it was fresh. Each set of expanded notes were discussed with fieldwork supervisors with the rapid implementation of any feedback.

*Analytical induction*, an iterative, inductive–deductive approach, was adopted as the analysis approach [31]. Interview and FGD topic guides were developed to explore barriers and facilitators of motivation, retention and performance as well as experiences and attitudes towards potential technology and community-based interventions. The original topic guides helped structure the thematic analysis, but scope remained for data to be generated in unanticipated content areas and for themes to emerge from the data [31]. *Expanded notes* from in-depth interviews and FGDs were analysed systematically for key themes using a content analysis approach [31-33].

## Results

The summary of the results of the theoretical review is followed by the results of the formative research. Both sets of results informed the identification of key implications for intervention design. These implications are presented following the theory and formative research subsections. A description of the two inSCALE interventions appears at the end of the results section.

### Theory

The results of the theoretical review relating to the broad area of *work motivation* (including its close relationship with retention and performance) are presented first before *CHW motivation* specifically and the selected theory—*the Social Identity Approach*.

#### Work motivation, retention and performance

The bulk of work motivation theory has been developed in high-income settings. In the early part of the twentieth century, enquiry into work motivation, and indeed retention and performance, typically rested on assumptions that behaviour was subject to a rational decision-making process occurring within the individual based on the pros and cons of a certain action or actions—i.e. ‘rational’ or ‘economic choice’ models [34-36]. Since the 1970s, from a social psychological perspective, Bandura’s social cognitive theory has been one of the dominant theories applicable to work motivation [35,36]. Social cognition theory seeks to explain ‘social behaviour with reference to individual mental processes’ (Hepburn, 2003, P. 19) [37], with *self-efficacy*, *outcome expectancies* and *goal congruence* as important concepts [36,38]. It follows that providing a strong incentive to reach a work target or goal will only result in a worker mobilising extra effort to achieve it if they believe it is attainable through their endeavours (self-efficacy), it is worth pursuing in terms of expected outcomes (outcome expectancies) and the work goal aligns with their personal goals (goal congruence) [36].

A contrasting theory, put forward by Kanfer and Heggestad (1999), is that work motivation occurs as a function of the interaction between the person and the situation [39]. A key component of this interface has been proposed as the degree to which a worker’s needs are satisfied—a factor commonly linked in the literature to the likelihood of their retention [10,11,18,35,36]. In the context of incentives, the power of a given incentive—such as a certain level of pay—to exact an increased level of performance from a worker has been seen as a function of both the degree to which the incentive is perceived to satisfy a worker’s needs and how important the satisfaction of those needs is to the worker.

Needs satisfaction theories have typically driven the provision by employers of the appropriate work conditions and tools for role performance and maintenance (i.e. retention). Along with social cognition approaches, this strand of motivational theory has been particularly influential in the context of CHW motivation where the focus has predominantly been on finding the right match between incentives and the individual worker. This focus has seldom however been accompanied by scrutiny of the impact of social or contextual factors on the generation of these needs [10,17,21,40].

#### CHW motivation, retention and performance

Reviews of the motivation, retention and performance of CHWs in low-income settings have commonly focused on the provision of incentives [10,11,16-18]. They tend to emphasise the individual worker’s cognitive response to their working, social and cultural context. Employment conditions and other contextual factors have, on occasions, been accounted for but usually in terms of how they are perceived by the individual. Thus, motivation has typically been viewed as an individual cognitive process where incentives influence or appeal to internal (e.g. values) and environmental (e.g. individual attitudes to contextual factors) components [12,16,20] with retention and performance occurring as a behavioural consequence of the level of motivation and job satisfaction (i.e. absence of dissatisfaction) [9-11]. This influence in combination with a general approach informed by the social cognition perspective has led to CHW work motivation being commonly defined as the ‘individual’s degree of willingness to exert and maintain an effort towards organizational goals’ [12,16].

The model of health worker motivation developed by Kanfer and colleagues (2002 and 2004) is typical of approaches focusing on individual cognition [16,20]. It breaks down the determinants of health worker motivation into three layers—individual-level determinants, work context/organisational determinants and determinants resulting from broader society and culture. It assesses these layers from the standpoint of individual cognition in terms of what they mean for individual attitudes and behavioural intentions.

While offering key insights into the individual cognitive processes of motivation, models such as Franco’s (2002 and 2004), and later Chandler’s (2009), have been criticised for their overt focus on individual cognitions. For instance, Campbell and Scott (2011) argue that, when applied to CHWs, behavioural models that focus on individual cognition tend to underemphasise the impact of community influence on CHW programme outcomes [4]. Such accounts of CHW motivation suggest that understanding the influence of community context and culture beyond individual cognitions is critical when seeking insight into the constraints and facilitators of CHW motivation and the key to developing effective, context-specific strategies [4,9,10,21,41]. From this perspective, exploring the influence of community on the social norms and priority needs of CHWs becomes important when seeking to understand CHW motivation and how this leads to retention and performance [9,10,21].

Drawing on a theory that takes specific account of the social processes that inform needs prioritisation, and the value ascribed to incentives in context, would thus appear to be beneficial when seeking to develop effective interventions to motivate CHWs, satisfy them sufficiently that they remain in role, and perform in a manner aligned with the objectives of the CHW programme. Indeed, such an approach may both complement the more traditionally adopted social cognitive methods and provide a response to calls for greater focus on the influence of CHW working context.

#### The Social Identity Approach

The Social Identity Approach^b^ (SIA) is a collection of behavioural theories that account for both individual cognition and contextual factors [34,42]. The SIA is psychological in that it offers insight into the processes within an individual that determine behaviour [42,43]. Critically, it is also social as it demonstrates how these processes are dependent upon interpersonal relationships and group memberships and their perceived value and significance to the individual [42,44].

According to the SIA, when a given group identity is relevant to an individual, and one *categorises* (i.e. identifies) oneself as a group member, one’s behaviour becomes subject to perceived group social norms and what is seen to be in the group’s interests [44,45]. Establishment of the group’s ‘positive distinctiveness’ and status, standing and esteem become important drivers of the self-identifying group member’s behaviour [34,42,44,46-48]. In the context of work motivation, when workers define themselves more in terms of personal identity, it could be expected that individual motivators such as personal advancement or recognition may be more influential. When defining themselves in terms of social identity, motivators that impact on the groups one identifies with such as working conditions or status may be more influential [34]. The SIA is increasingly being applied in the context of work motivation—albeit more typically in high-income countries [34,43]—with this application yielding promising evidence for the link between identification and motivation [49-51].

It was determined by the inSCALE study group that the SIA provided a potentially valuable framework to guide the design of CHW interventions alongside key elements of the social cognition approach. We hypothesise that CHW motivation will increase as a function of the relevance of the CHW social identity to CHWs and the resulting identification of CHWs with that collective [34,48]. It is further proposed that work motivation is subject to the perception that the pursuit of the behaviours and activities required by the programme are in the collective’s best interests [48]. Thus, if actions that promote the positive distinctiveness of the CHW collective are clearly communicated and understood, it is proposed that CHWs identifying with a shared social identity will be motivated to perform those actions [34,48]. Positive performance and retention is likely to follow to the degree that these are consistent with CHW perceptions of effective performance (i.e. it is worth it) and within the control of CHWs to influence and potentially achieve [34,48,49]. Assessing the cost-effectiveness of interventions developed based on the SIA will be a critical aspect when exploring the potential for implementation at scale in Uganda and Mozambique.

### Key implications from the theoretical review for intervention design

From the results of the theoretical review, five key guiding principles for the intervention design were identified. These were that the interventions should:

1. Promote the correspondence between CHW goals and those of the programme.
2. Stimulate a feeling of shared experience and collective identity among CHWs.
3. Promote the value of achieving CHW programme goals to the CHW collective.
4. Clearly communicate the actions that lead to good and appropriate performance of CHW duties and promote the link between this performance and the distinctiveness of the CHW collective.
5. Focus on what is within the CHW’s power to deliver.

### Formative research

The formative research results are presented in thematic areas that most directly influenced the development of the interventions, namely *motivation*, *retention*, *performance*, *community issues and influence*, and *challenges*.

#### Motivation

In Uganda, VHTs reported being motivated by helping fellow community members, not wishing to let them down and gaining their trust, respect and appreciation. They also found learning, meeting new people and receiving validation and feedback from their supervisors motivating.

*When I began treating young people in the community by giving them drugs it has earned me respect in the community … this makes me feel motivated and feel good* (U VHT IDI 14)^c^.

The benefit of having access to drugs to treat their own children was an added incentive.

In Mozambique, APEs reported being motivated by the responsibility of being chosen by their communities and the respect afforded them as someone perceived to be doing important work that contributes to a healthier community. They felt responsible for making best use of the training provided to them by virtue of being their community’s representative to bring better health outcomes to the community.

*I feel very well in doing my work because I have been chosen by the community members to help them, the people trust in me* (M APE IDI 12).

#### Retention

VHTs did not generally anticipate leaving their role though did on some occasions suggest that they would leave if they felt they had lost community trust and appreciation. They did commonly note however that events such as moving villages, falling ill or being voted out as the community representative could lead to them leaving as could being offered a paid role.

*If I get a job where I am paid some money I would leave the VHT work because my family needs to survive and VHT work is voluntary* (M VHT IDI 9).

APEs and supervisors suggested that they would not leave their role and could not anticipate circumstances where this would be necessary. Commonly, a sense of duty and calling was cited as the reason.

*As a Mozambican citizen I have to help the sick people … I will never stop working as an APE* (M APE IDI 11).

#### Performance

Discussions related to VHT performance centred on the amount of technical support provided, the need for health-related information and rapid feedback on submitted data. Such feedback, it was suggested, could come from the health facility-based supervisor or the community as represented by a committee.

*I think it would be better in case there is a committee of people who can monitor the performance of these VHTs and also know their challenges and in case of any problems they can report to these people because as you know, people these days are not trusted, in case they are given medicine, they can decide to sell it so when there is a team monitoring these VHTs, they will fear to do so* (U Community Leader IDI 5).

*Once I am told about how I perform it will motivate me to keep up the good performance or if I am performing below standard I will work hard to be a better performer* (U VHT IDI 31).

APEs commonly saw the value of supervision and support networks and identified the link between this and their performance on specific work tasks.

*When I face a problem with my work I have sought the help of my supervisor because she is the person responsible for me and any problem that I might have and I cannot solve it alone she is the person who can help me* (M APE IDI 3).

For APEs, performance was heavily influenced by the availability of resources—especially drugs and transport means—as it impacted on the ability to treat patients. For VHTs, this challenge was felt to also impact on local credibility.

*I’ve always reported to my supervisor about the problems of lack of money to pay for transportation for submitting data and collecting the medicines at the health facility but this concern has not yet solved* (M APE IDI 10).

*I think the problems VHTs face are the drugs get out of stock and this interrupts the service they give* (U Male Head of Household IDI 2).

In Mozambique, while some supervisors felt APEs experienced challenges with their performance and emphasised the need for greater levels of technical supervision, others suggested that APEs had few problems. Supervisor perspective may however be influenced by their limited view of APE work due to the challenge of travelling to meet the APEs they supervise.

*My interaction with the APE depends on what we have to do. But it has not been regular as I would like… sometimes the APEs themselves call to me or I call them to ask for some specific information. For example, when we have a province visit from the APEs provincial programme staff, we take the opportunity to discuss issues related to our work* (M Supervisor IDI 4).

#### Community issues and influence

In Uganda, the community and VHT response to proposed participatory activities with community members indicated that over time those that are invested in positive health outcomes in their community will maintain their efforts if content is fresh and locally meaningful and activities are purposeful and enjoyable. Community groups have reportedly met resistance in the past due to perceived agendas, exclusivity and inaction which have in turn led to apathy towards organised community meetings.

*I normally notice a major problem of people losing trust and hope in these groups especially when their expectations are not met … they end up demoralised as a result of no actions* (U Caregiver to child below 5 years of age IDI 2).

Promoting the role of the VHT and the benefits they bring to community members as part of the solution to health challenges rather than emphasising health problems was suggested when seeking to maintain community participation and support of VHTs and increase VHT motivation.

*If the VHT sees that the people s/he serves are concerned they will be more committed and love their work more* (U Supervisor IDI 1).

*I can easily explain to the members the challenges I experience and would also get their own views and ideas and together we can get a way forward….. it creates a level of understanding and togetherness* (U VHT IDI 3).

In addition, VHTs saw the value of visible signifiers of their role as VHTs in generating community esteem.

*Even having a VHT marked phone brings you respect from the people in your community* (U VHT FGD 6).

In Mozambique, community members and APEs reported a good relationship between APEs and the community. APEs value the support/relationship and care how they are viewed by the community. The community use the APEs, think their work is important and respect them.

*I feel very well in doing my work because I have been chosen by the community members to help them, the people trust in me and recognise that I have been doing a great work in community* [sic] (M APE IDI 12).

As a result, while participatory activities in the community were not proposed in Mozambique, the intervention should highlight community support and use terminology that APEs find meaningful such as community reputation, respect and recognition.

#### Challenges

In Uganda, VHTs experienced some challenges with members of their local community based on what they saw as a misunderstanding of their role. They identified endorsement by supervisors as contributing to improved community credibility. VHTs also reported drug and essential equipment stock outs as well as challenges related to the distance from the health facility and their supervisors.

*Community trust is increased by seeing our supervisor … local authority interaction with VHTs would show they are valued* (U VHT IDI 13).

As in Uganda, APEs in Mozambique on occasion faced the challenge of a lack of community understanding of the purpose and scope of their work. They felt that more frequent support and supervision for technical challenges was desirable and may aid their local credibility but identified the distance to their supervisors and the health facility as representing an obstacle to this occurring.

*I have had contact with my supervisor because sometimes she has been visiting me here in the community to see how I’m taking care of sick children and to know what are my problems… but that was last year, this year she has not visited me yet* (M APE IDI 9).

### Key implications from the formative research for intervention design

From the results of the formative research, six key guiding principles for the intervention design were identified. These were:

1. CHWs are motivated by their status and standing in the community and a sense of the value they add.
2. CHWs value technical feedback and supportive encouragement from both supervisors and community members.
3. Feeling connected to both the health system and the community they serve motivates and validates CHWs in their role.
4. Adequate resources—especially drugs—must be in place for CHWs to be motivated and perform.
5. Participatory activities in the community that are open to all, enjoyable, purposeful and focused on positive local health outcomes delivered through the CHWs are likely to sustain community interest and engagement and be motivating for CHWs (in Uganda).
6. Interventions supported by ICT that facilitate easy communication, provide context-specific technical support and engender a sense of connectedness to the health system, supervisors and peers are feasible and acceptable to CHWs in Uganda and Mozambique and likely to increase CHW motivation.

### Interventions

Based on the key implications for intervention design drawn from the theoretical review and formative research, and following the development process outlined in Table 1, a ‘community-supported intervention’ was developed in Uganda and a ‘technology-supported intervention’ in Uganda and Mozambique. Drawing on social cognition approaches and the SIA specifically, as well as the results of the formative research, both interventions were designed to appeal to CHW goals through emphasising status and standing, promoting connectedness to the health system and the community, and providing technical feedback, encouragement and resources.

Based on the finding from the formative research that purposeful and participatory community activities that are open to all were likely to be motivating and sustainable for CHWs, the establishment of village health clubs [52] were decided upon as the key activity in what is called the ‘community-supported intervention’ in Uganda. Village health clubs will provide community access to technical and local health knowledge through CHW-facilitated meetings where attitudes to child health and the enabling role of the CHW can be positively influenced and realistic expectations promoted. The clubs will encourage CHW collaboration with and accountability to fellow community members in the shared enterprise of improved community health through the implementation of an action planning cycle. In doing so, it is proposed that community appreciation, understanding and respect for CHWs as well as a sense of community connectedness, all identified through the formative research as important to CHWs and their motivation, will increase. As a result, CHWs will, it is proposed, more readily identify themselves as a member of a CHW collective. See Table 3 for how the main findings from the theoretical review and formative research support the key elements of the community intervention.

**Table 3:** **Emphases from the theoretical review and formative research that support the key components of the community intervention**

**Community intervention**		
**Intervention**	**Way in which intervention will improve CHW motivation**	
	**Theory**	**Formative research—Uganda**
Participatory village health club (VHC) facilitated by a CHW that is open to all, fun and focused on local health improvement *via* local community assets with emphasis on the CHW	• Reinforcement and validation of CHW role value to CHWs through facilitation of the VHC and receiving community feedback	• CHWs want a greater sense of connectedness to their community. Community groups established to monitor and provide feedback to CHWs may improve motivation and performance by bringing CHWs closer to their community
	• By directly seeing and receiving feedback on impact of their work, CHWs will more readily recognise the value of their work to the community and CHW collective	• Promoting the positive work that CHWs do in their community is reportedly motivating for CHWs and may engender greater community trust and standing/status of the CHWs
	• Working directly with community members as they identify, prioritise and find solutions to local health challenges will reinforce a sense of connectedness between CHWs and their community	• CHWs value community feedback
	• By operating in an interactive local forum, community expectations around what it is within the CHWs’ power to deliver can be explained and managed	• Locally meaningful activities are more likely to be sustained by the community with community groups highlighting the positive role played by CHWs, improving community esteem for CHWs

The second intervention, known as the ‘technology-supported intervention’ will provide CHWs with mobile phones and solar chargers as both key tools for CHW work and signifiers of role that are likely to confer status and standing as indicated by the formative research results. Linking to peers and supervisors through either calls or *short message service* (SMS) on the provided phones where the content of the communication is to be self-determined is a key component of the intervention that aims to promote opportunities for supportive encouragement as well a sense of connectedness to both the CHW community and the health system. Rapid, context-based feedback on submitted data is designed to both promote a sense of relevance of CHW work and validate each CHW’s contribution to a health system of which they are a member. Regular SMS-based technical and motivational messages will address CHWs’ identified need for technical feedback and support and represent an opportunity for the promotion of CHWs as key members of a valued and important collective. In addition, the supplied phones are programmed with software designed to directly influence role performance such as modified respiratory timers in Uganda and electronic job aids to support patient consultation in Mozambique. See Table 4 for how the main findings from the theoretical review and formative research support the key elements of the technology intervention.

**Table 4:** **Emphases from the theoretical review and formative research that support the key components of the technology intervention**

**Technology intervention**			
**Intervention component**	**Way in which intervention will improve CHW motivation**		
	**Theory**	**Formative research**	
		**Uganda**	**Mozambique**
Closed user groups	Potentially stimulate a feeling of shared experience and collective identity among CHWs.	Considered feasible and acceptable by Ministry of Health personnel	
		Phone as a signifier of role—may increase the status and standing of CHWs in their community	
		Increase ease of communication with supervisors and promote a sense of connectedness to health system	
	Specifically, by promoting interaction with peers and supervisors, CHWs may:	Aid prompt CHW reporting of stock outs and other challenges	
		No need for CHWs to use own phone—cost saving plus potential to earn from solar charger	
		CHWs already meet informally so formalises an existing structure that is valued	
	• Gain a greater sense of the correspondence between CHW goals and those of the programme	Need for guidance on who initiates calls as CHWs can find unscheduled calls stressful	Concerns around supervisor workload led to recommendation that supervisors be available for contact at certain times only potentially leading to the provision of more efficient and targeted feedback through managing supervisor workload
	• Understand the value of achieving programme goals to the community and CHW collective		
	• Understand and normalise positive, appropriate and distinguishing actions of ‘good’ CHWs		
CHW electronic data submission and feedback and targeted supervision	Through targeted feedback delivered by supervisors:	Feeling valued and linked to the health system	
		Feeling encouraged by positive local gains/improved community health and their role	
	• Gain a greater sense of the correspondence between own goals and those of the programme • Understand and normalise positive, appropriate and distinguishing actions of good CHWs • Promote realistic actions of CHWs that are within their power to deliver	Strong desire among CHWs for feedback and more supervision—targeted supervision welcome	
		Concerns about supervisor speed of responsiveness—needs to be sufficiently prompt to avoid CHW discouragement	Tone of messages key with need for polite and respectful language emphasised in order to be motivating with no admonishments for poor performance
Monthly motivational SMS	Through contextually appropriate and regular messages:	Positive, encouraging, polite and respectful tone with emphasis on the value CHWs bring to their community	
		Important to feel valued, supported and linked to the health system	
	• Promote the correspondence between CHW goals and those of the programme	Receiving messages that are locally relevant and address key challenges promotes a sense of CHW relevance and importance to the health system	
		If the message resonates with data submitted, then will be perceived as performance-related feedback which was considered motivating by CHWs	
	• Promote the shared experience of CHWs		
	• Promote the value of achieving programme goals to the CHW collective		
	• Validate and normalise positive, appropriate and distinguishing actions of ‘good’ CHWs		
	• Promote realistic actions of CHWs that are within their power to deliver		

While the interventions were designed based on a number of theories in addition to the SIA (notably outcome expectancies, goal congruence and an emphasis on self-efficacy from the social cognition theoretical tradition), the SIA is being explicitly described as a new and novel addition to the current theoretical literature relating to CHWs. As a result, both the community- and technology-supported interventions seek to promote positive CHW identity in a manner that resonates with the stated priorities of CHWs. The interventions are also designed to communicate the actions CHWs can take in carrying out their duties that will maintain this positive identity. It is proposed that CHWs will be more motivated to carry out these duties in intervention areas when compared against a control group.

## Discussion

The inSCALE project utilised behavioural theory and the findings from formative research when developing two interventions to address the challenge of CHW motivation, retention and performance in Uganda and Mozambique. The findings of the theoretical review led to an emphasis during the intervention design process on the promotion of synergies between CHW goals and those of the CHW programme, the value of achieving those goals and building a sense of collective CHW identity. In addition, it was proposed that if the interventions could communicate desirable behaviours compatible with the CHW role as designed that were also perceived to improve the esteem in which CHWs were held, socially identified CHWs would be motivated to adopt these behaviours and performance benefits would follow.

Formative research conducted with CHWs and stakeholders in CHW programmes also strongly influenced intervention design. The formative research findings led to a specific emphasis during the intervention design process on generating feedback from the health system and the community as well as cultivating a sense of connectedness with both. Promoting CHW status and standing was an additional emphasis. While broadly consistent with the recommendations of other studies of CHW motivation, the formative research revealed that these emphases were the most feasible and acceptable approaches in the context and were thus preferred to alternatives among a suite of strategies proposed elsewhere [9,10].

The inSCALE project is currently evaluating the effectiveness of the interventions. The perceptions of the main stakeholders (including CHWs) regarding the barriers to and facilitators of successful implementation will be explored qualitatively. Motivation, performance and retention as well as social identification of CHWs will also be assessed quantitatively through the development of appropriate questionnaires and the adoption of a cluster randomised control trial design. Drug supply and supervision will be controlled. Importantly, the different operating contexts CHWs face in Uganda and Mozambique will be accounted for when comparing results from the two sites and drawing conclusions regarding the utility of the theory and the effectiveness of the interventions when seeking to influence CHW motivation, performance and retention. The first aim of the evaluation is to provide insight into what influences these key constraints to the delivery of services utilising cadres of CHWs. As a second aim, the data generated will allow for an analysis of the suitability of the SIA as a behavioural theory to guide intervention development in these settings. A third aim is to contribute to the evidence base for the effectiveness of interventions targeting CHW motivation that is currently small in the case of both community participatory and technology-supported interventions.

**Figure 1:**
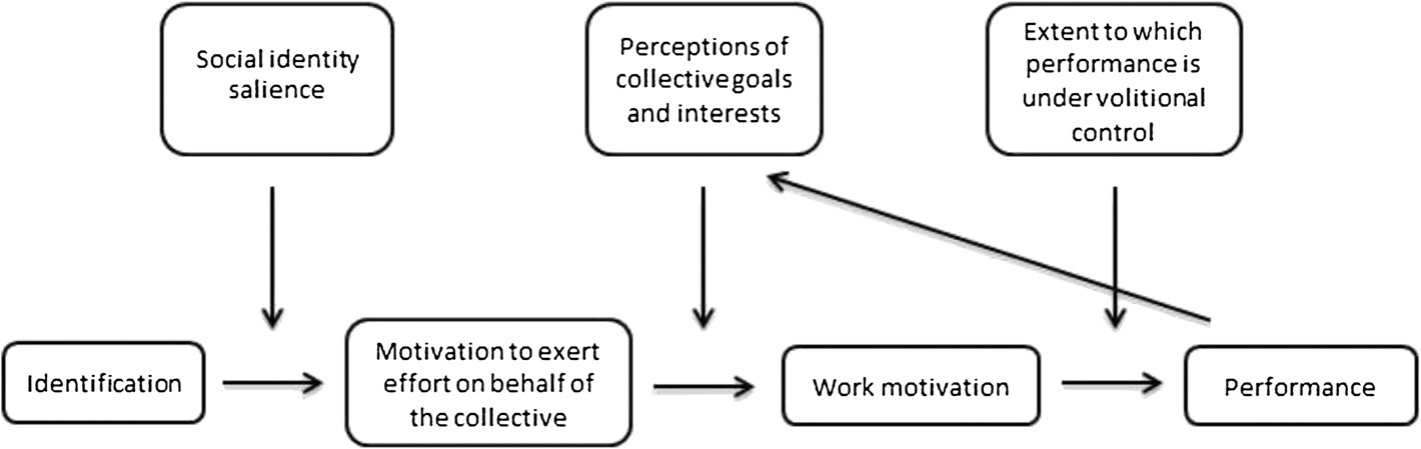
A social identity model of work motivation and performance (van Knippenberg, 2000) [48]. © International Association for Applied Psychology, 2000^d^.

For the purpose of assessing the SIA for suitability when designing CHW motivation interventions, a model developed by van Knippenberg (2000) [48] will be drawn upon (Figure 1). When applying the model to CHWs it proposes that CHW satisfaction with their role and motivation to exert effort on behalf of the CHW programme is a function of the salience or relevance of the CHW social identity to CHWs and resulting identification with that collective. It further proposes that work motivation is subject to the perception that the performance of the work-based behaviour required by the programme is in the collective’s (i.e. CHW’s) best interests. Thus, if the actions that promote the positive distinctiveness of the CHW collective are made clear by the programme environment (i.e. current CHW programme plus the inSCALE interventions in the current study), CHWs identifying with a shared social identity will, it is proposed, be motivated to perform those tasks.

The SIA was deemed by the inSCALE study group to represent an innovative approach to the enduring challenge of CHW motivation, retention and performance. There are however many behavioural theories, and a case could be made for the adoption of alternatives to the SIA. The SIA was selected primarily in response to the call for greater emphasis and sensitivity to the context of CHW work [4] which ruled out a focus on, for instance, personality—an influential line of work motivation enquiry in the twentieth century not explored in this paper [35]. The selection of the SIA was made by an experienced multi-country team tasked with being innovative and was based on several reviews and consultations [9,23].

The inSCALE study group determined that if a link can be established between CHW motivation and the identification of individual CHWs with a collective CHW identity, then there may be a rationale for the development of simple, collective identity-focused interventions when seeking to influence CHW motivation at scale. Indeed, a focus on the development of interventions that appeal to the needs of CHW collectives in different contexts, and thus galvanise them *en masse* to perform the actions they perceive to be in the interests of the group with which they identify, may offer a cost-effective complement to the traditional strategy of creating an incentive package [10,11,16-19].

## Conclusion

It is proposed in this paper that drawing on contextual data and theory that is sensitive to context will lead to the development of more appropriate and effective interventions when aiming to improve the motivation, retention and performance of CHWs in Uganda and Mozambique. Evaluation of the interventions developed by the inSCALE project and described in this paper will allow for an assessment of the suitability of the SIA for guiding intervention development for this purpose. In addition, it will inform the assessment of whether taking interventions designed drawing on its key tenets to national scale in Uganda and Mozambique is warranted. Should this prove to be the case, an increased focus on appealing to the needs of socially identified collectives of CHWs may represent a relatively simple, cost-effective and complementary strategy to the traditional approach of tailored incentive packages.

## Endnotes

^a^Written consent was obtained from all research participants before interview. The trial protocol was approved by Makerere University Institutional Review Board in Uganda, the Uganda National Council of Science and Technology (ref. HS 958), the Comité Nacional de Bioética para a Saúde in Mozambique (ref. 331/CNBS/12) and London School of Hygiene & Tropical Medicine Ethics Committee in the UK (ref. 5762). The study has been registered as a randomised controlled trial with http://www.clinicaltrials.gov (identifier NCT01972321).

^b^The Social Identity Approach was a term coined to describe social identity theory and social categorisation theory and the bank of empirical work and evidence-based practices that have been developed through the 30 or so years of the approach’s development [34].

^c^NB: The results are presented with direct illustrative quotes from respondents. They are followed by abbreviations to indicate from which country and group they originated (U = Uganda, M = Mozambique, VHT = Village Health Team member in Uganda, APE = Agente Polivalente Elementar in Mozambique, supervisor, male head of household, caregiver for child below 5 years of age, community leader) and the number of the IDI or FGD.

^d^© International Association for Applied Psychology, 2000. Published by Blackwell Publishers, 108 Cowley Road, Oxford OX4 1JF, UK and 350 Main Street, Malden, MA 02148, USA.

## Abbreviations

APE: Agente Polivalente Elementar
CBA: Community-based agent
CHW: Community health worker
FGD: Focus group discussion
ICCM: Integrated Community Case Management
ICT: Information and communication technologies
IDI: In-depth interview
inSCALE: Innovations at Scale for Community Access and Lasting Effects
SIA: Social Identity Approach
SMS: Short message service
WHO: World Health Organization
